# *Hirschioporus abietinus* Laccase: Cloning, Heterologous Expression, Characterization and Solvent Tolerance Evaluation

**DOI:** 10.3390/molecules31030458

**Published:** 2026-01-28

**Authors:** Ingrida Radveikienė, Marius Dagys, Rita Meškienė, Rolandas Meškys, Regina Vidžiūnaitė, Vida Časaitė

**Affiliations:** 1 Department of Bioanalysis, Institute of Biochemistry, Life Sciences Center, Vilnius University, Sauletekio Ave. 7, 10257 Vilnius, Lithuania; i.radveikiene@gmail.com (I.R.); marius.dagys@gmc.vu.lt (M.D.);; 2 Department of Molecular Microbiology and Biotechnology, Institute of Biochemistry, Life Sciences Center, Vilnius University, Sauletekio Ave. 7, 10257 Vilnius, Lithuania; rita.meskiene@bchi.vu.lt (R.M.); rolandas.meskys@bchi.vu.lt (R.M.)

**Keywords:** laccase, *Hirschioporus abietinus*, *Pichia pastoris*, organic solvent

## Abstract

Laccases are versatile biocatalysts with broad industrial relevance. Their heterologous expression enables efficient production, purification, and functional optimization. The white-rot fungus *Hirschioporus abietinus* produces an effective extracellular laccase (Lac2), inspiring the identification and cloning of its encoding gene. To enable high and stable enzyme production, the gene was expressed in *Pichia pastoris* and the cultivation conditions for the selected variant were optimized to enhance the yield of recombinant laccase. The Lac2 was then purified and its biochemical properties characterized. The high-redox potential laccase Lac2 exhibited strong tolerance to common metal ions and maintained catalytic activity in the presence of a range of organic solvents. Overall, the results suggest that Lac2 possesses properties compatible with small-scale production and effective use in biosensor systems.

## 1. Introduction

Laccases (benzenediol: oxygen oxidoreductases, EC 1.10.3.2) are a family of multi-copper oxidoreductases that can oxidize a wide range of aromatic compounds while reducing molecular oxygen to water [1]. Laccase is classified as a green enzyme because its catalytic activity is driven by the presence of four copper atoms at its redox-active sites, in contrast to peroxidases and monooxygenases that rely on hydrogen peroxide or reducing cofactors such as NAD(P)H [2]. Laccases are widely distributed in nature and are produced by bacteria, fungi, insects, and plants, with fungal laccases being the most extensively studied. At present, more than 100 laccases from basidiomycete and ascomycete fungi have been purified and characterized [3,4,5,6,7]. Additionally, many laccase genes have been cloned. To date, approximately 50 expression studies have been successfully carried out using various expression systems, including *E. coli*, *Kluyveromyces lactis*, *Saccharomyces cerevisiae*, *Yarrowia lipolytica*, *Pichia* spp., *Aspergillus* spp., and transgenic maize [8,9]. More than 20 fungal laccases have been expressed in yeasts such as *Pichia pastoris* and *Saccharomyces cerevisiae* for use in environmental and industrial applications [10]. In general terms, both organisms are suitable for expressing eukaryotic genes. The *P. pastoris* system has been largely used to express proteins that require post-translational modifications (disulphide bridge formation, C- and N-terminal processing, and glycosylation). This host is easily manipulated due to the availability of a large set of molecular biology tools. When grown on minimal medium, *P. pastoris* secretes recombinant proteins in the presence of low levels of native proteins, which facilitates the recovery and purification of the target protein [11].

Laccases have been used in many processes such as delignification of lignocellulose compounds [12], bio-pulping and bio-bleaching [13], transformation of colourants in the textile industry [14], wastewater treatment [15], and degradation of explosives [16] and pesticides [17]. Additionally, they can be utilized in the development of biosensors for the clinical and environmental analysis of carbohydrates [18,19], oxygen [20], azides [21], morphine [22], codeine [23], catecholamines [24], flavonoids [25], etc. [10,26].

Certain laccase substrates, including complex lignin derivatives, aromatic amines, phenols, flavonoids, and a variety of persistent organic pollutants, are insoluble or only minimally soluble in water. The solubility may be enhanced by adding organic solvents to reaction mixtures, but the wide application of laccases limits their sensitivity to organic solvents. In part, these limitations can be overcome using organic solvent-tolerant enzymes. Laccases have recently been shown to be active in reaction media where most of the water has been replaced by an organic solvent [27,28,29,30]. Laccases, stable and active in the presence of organic solvents, are a very promising tool for biotechnological applications, such as bio-bleaching, detoxification of hazardous compounds present in wastewater, and organic synthesis [30,31,32].

*Hirschioporus abietinus* (formerly *Trichaptum abietinum*) is a common wood-decaying fungal species that produces a pulpy white rot. Due to their ligninolytic activity, particularly the involvement of laccase enzymes in lignin degradation, white-rot fungi have promising applications in bioremediation and the breakdown of certain plastic polymers [33,34,35]. In our previous research, we isolated a laccase from *H. abietinum* and demonstrated its suitability for bioelectrocatalytic oxygen reduction based on direct electron transfer [36,37,38]. The aim of this study was to identify the gene encoding this laccase, thereby supporting more efficient enzyme purification and enabling future genetic modification. The laccase gene of *H. abietinus* Lac2 and its full-length cDNA were cloned and characterized. The 1560 bp full-length cDNA, which encodes an intact open reading frame (ORF), was expressed in *P. pastoris* cells, purified, and characterized in terms of its biochemical and catalytic properties. We also investigated the ability of Lac2 to function in the presence of organic solvents.

## 2. Results

### 2.1. Laccase Gene of H. abietinus

To identify the laccase gene of *H. abietinus*, we determined the amino acid sequences of tryptic peptides of the native laccase purified from medium as described previously [36,37] using MS-MS. Based on the obtained sequence and conserved copper-binding regions, we designed gene-specific primers. PCR amplification yielded two DNA fragments of approximately 300 bp and 1500 bp, which were subsequently sequenced. Reverse PCR, performed as described in the Section 4, allowed us to determine the start and end points of the gene. Using these data, we amplified the full-length laccase gene from both genomic DNA and cDNA; this laccase was designated Lac2.

The cDNA was 1560 bp in length and corresponded to a 2799 bp genomic sequence. The open reading frame encoded a predicted 520-amino acid protein with a theoretical molecular mass of 55.8 kDa. Comparison of the genomic and cDNA sequences revealed 18 exons, with introns ranging from 55 to 70 bp. All intron splice junctions corresponded to the canonical GT/AG rule. The deduced protein contained a 21-amino acid signal peptide with the characteristic structure of a secretory signal sequence, including a positively charged amino terminus, a hydrophobic core, and small residues.

**Figure 1:**
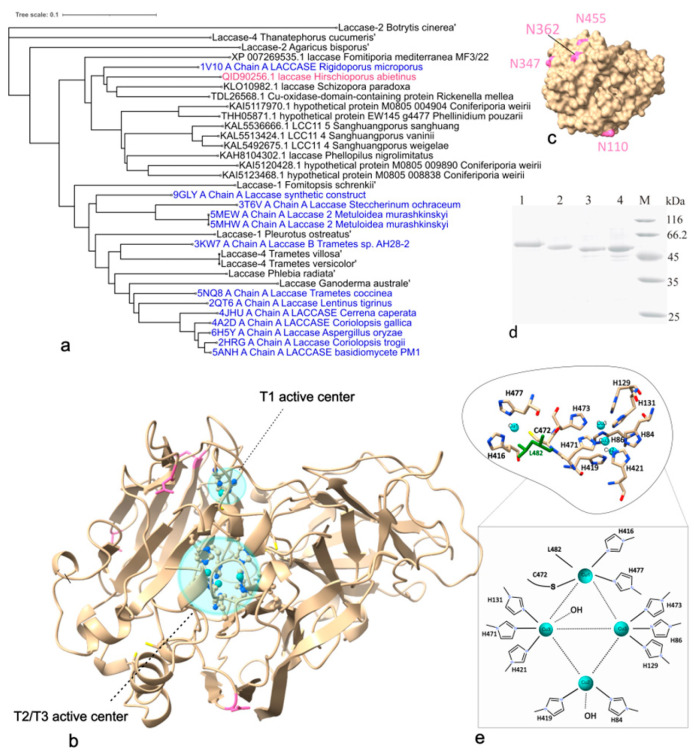
Analysis of Lac2. (**a**) Phylogenetic analysis. The phylogenetic tree was constructed using the Neighbour-Joining method and MEGA 7.0 software. The bar represents 0.1 substitutions per amino acid site. *H. abietinus* Lac2 is indicated in magenta; sequences from the PDB are indicated in blue; and sequences from the Swissprot-Uniprot/NCBI NR databases are indicated in black. (**b**) The AlfaFold2 model of Lac2, showing the active centres in cyan and the hypothetical N-glycosylation sites in magenta. The amino acids involved in the active centre are shown as a ball-and-stick model, and the copper ions are depicted as turquoise spheres. (**c**) The surface of Lac2 with indicated (magenta) glycosylation sites. (**d**) Analysis of the glycosylation of Lac2 (1—recombinant Lac2, 2—native Lac2, 3—recombinant Lac2 after treatment with endoglycosidase H, 4—native Lac2 after treatment with endoglycosidase H, and M—molecular weight marker). (**e**) The enlarged amino acid residues of the laccase active site and the schematic representation of the active site. The amino acids involved in the active site are represented as stick model and the copper ions are shown as turquoise spheres.

Typical fungal laccases are glycosylated proteins. The Lac2 sequence contained four potential N-glycosylation motifs (Asn–Xxx–Ser/Thr) at positions 110, 347, 362, and 455 of the deduced amino acid sequence (Figure 1). AlphaFold2 modelling indicated that all four asparagine residues are surface-exposed. Sequence analysis also identified three cupredoxin-like domains, the characteristic architectural feature of fungal laccases [39,40].

Ten histidine residues and one cysteine residue—required for coordinating the four copper atoms at the three canonical Cu(II) centres—were identified in the deduced Lac2 amino acid sequence. A total of five cysteine residues were present. Based on comparison with other characterized laccases and structural modelling, Cys472 was identified as the ligand to the type 1 copper site. Two disulphide bridges were predicted, formed by the pairs Cys105–Cys507 and Cys137–Cys230, both of which are conserved among most fungal laccases.

Sequence comparisons with laccases available in public databases showed that *H. abietinus* Lac2 shares 60–72% identity with laccases from species within the Hymenochaetales and Polyporales orders of Agaricomycetes. The highest similarity was observed with a *Rickenella mellea* Cu-oxidase domain-containing protein (Figure 1). Among structurally characterized laccases in the PDB, the closest match was the laccase from *Rigidoporus microporus* [41].

### 2.2. Expression of Lac2 in P. pastoris

To investigate Lac2 expression in *P. pastoris*, we constructed the pHC-lac2-4 expression plasmid. Laccase expression was driven by the methanol-inducible AOX1 promoter, and the gene was expressed with its native signal peptide and a CYC1 transcription terminator. Laccase activity became detectable on the second day of cultivation and reached a maximum of ~6000 U/L after twelve days. The highest activity was observed in the PichiaPink™ Strain 1. The activity was about four-fold lower without copper salts in the medium. Adding copper to the medium restores activity, indicating that the recombinant enzyme is produced in its apo form. (Supplementary Figure S1).

**Figure 2:**
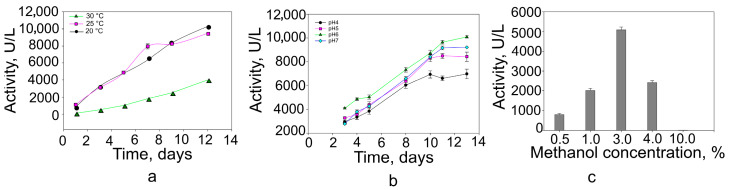
Production of extracellular recombinant laccase. (**a**) Effect of growth in temperature, (**b**) effect of medium pH, and (**c**) effect of methanol concentration (after 7 days of incubation) on the secretion of recombinant Lac2.

To optimize production, we examined the effects of temperature, pH, and methanol concentration on recombinant laccase expression. Lower cultivation temperatures favoured enzyme production (Figure 2). Optimal activity was obtained at pH 6.0, whereas pH 4.0, 5.0, and 7.0 resulted in markedly reduced activity. Cultures were induced with 0.5, 1, 3, 4, or 10% (*v*/*v*) methanol; laccase activity increased with methanol concentration, reaching a maximum at 3% (*v*/*v*). Under optimized expression conditions, laccase activity in the culture medium reached approximately 10,000 U/L.

### 2.3. Characteristics of the Recombinant Laccase

Lac2 was produced under optimized culture conditions and subsequently purified. SDS–PAGE analysis revealed a single band of approximately 66 kDa, higher than the theoretical molecular mass of 55.7 kDa. Following treatment with endoglycosidase H (Endo H), the apparent molecular weight decreased from 66 to 59 kDa, confirming that the recombinant Lac2 had glycosylated. Although the native *H. abietinus* laccase exhibited a lower apparent molecular weight than the recombinant enzyme, both proteins displayed identical molecular weights after deglycosylation (Figure 1d). Laccase was purified with an activity yield of 32%, and the resulting enzyme exhibited a specific activity of 600 U mg^−1^. Maximum (interval) volumetric productivity was 950 U/L/day.

The catalytic properties of the purified laccase were examined with various phenolic and nonphenolic compounds, such as monoaromatic phenolic substrate (2,6-DMP), a complex phenol (SGZ), a nonphenolic heterocyclic compound (ABTS), a phenoxazine ring-containing compound (PPA), a phenothiazine ring compound (PZ), and an inorganic compound (K_4_Fe(CN)_6_) (Table 1). The lowest K_M_ value for Lac2 was with PPA, which indicates the highest affinity for this substrate. Also, there was high affinity (K_m_ = 5.5 ± 0.7 µM and k_cat_/K_M_ 102 ± 3.0 µM^–1^·s^–1^) with K_4_Fe(CN)_6_. The lowest catalytic efficiency was obtained with CAT followed by HQ.

**Figure 3:**
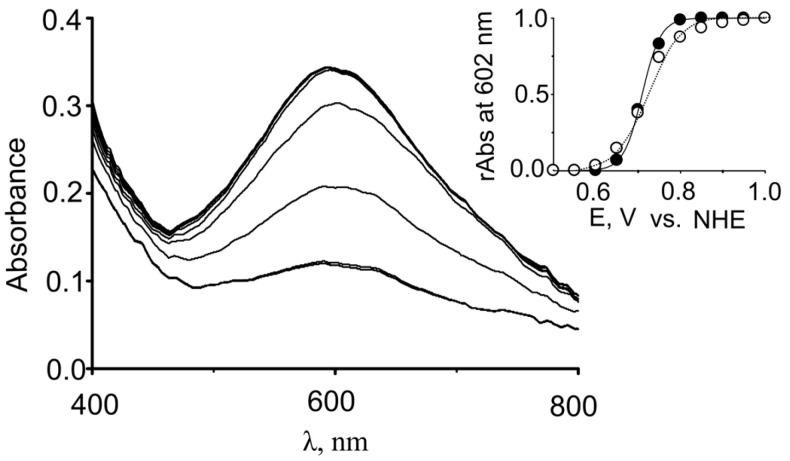
Redox titration of the T1 copper site in Lac2 using the redox mediator K_4_[Mo(CN)_8_]. The insert shows the dependence of the relative absorption at 602 nm on the electrochemical cell potential. The model described by Equations (1) and (2) is plotted over the data points. The solid line represents the oxidative scan direction and the dotted line represents the reductive scan direction.

Lac2 of *H. abietinus* was observed to have a redox potential (E_Redox_) of 0.714 V for the type 1 Cu (T1) site (Table 2 and Figure 3). Hence, Lac2 seems to belong to a group of high-redox potential laccases.

**Figure 4:**
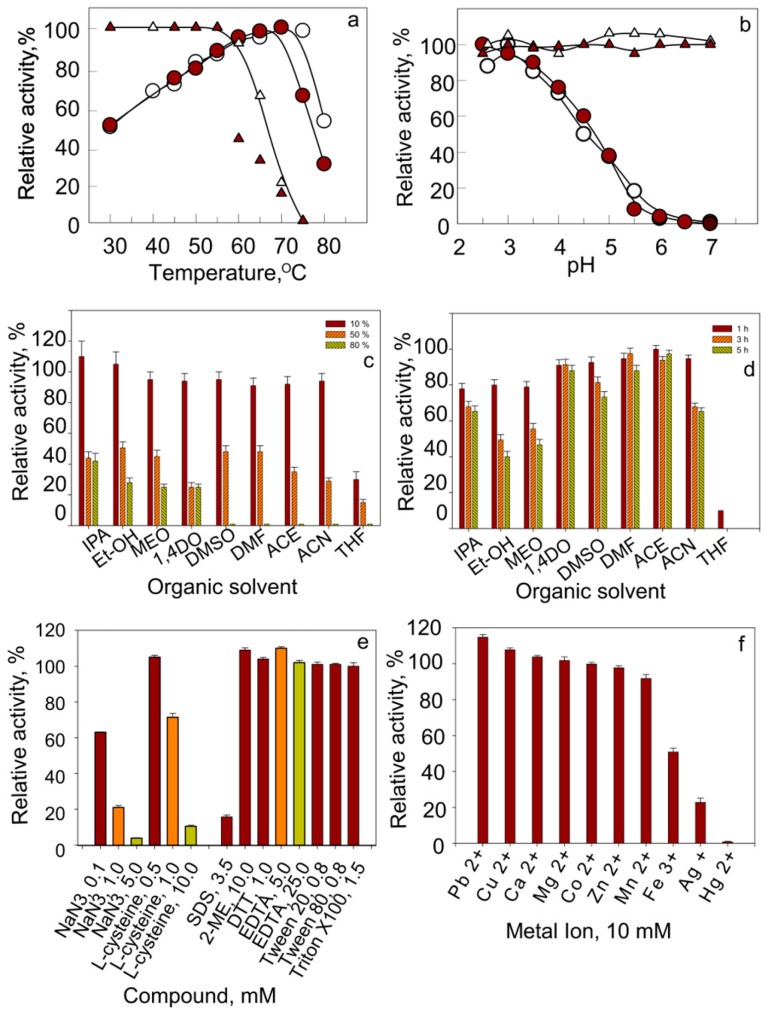
Biochemical properties of Lac2. (**a**) Temperature-dependent activity (circles) and thermal stability (triangles) of native (open symbols) and recombinant (closed symbols) Lac2. (**b**) Effect of pH on the activity (circles) and stability (triangles) of native (open symbols) and recombinant (closed symbols) Lac2. (**c**) Relative activity of Lac2 in the presence of various organic solvents at 10, 50, and 80% (*v*/*v*). (**d**) Stability of Lac2 after incubation in organic solvents for 1, 3, and 5 h. (**e**) Relative activity of Lac2 in the presence of common laccase inhibitors at the indicated concentrations. (**f**) Effect of 10 mM metal ions on the activity of purified Lac2. Abbreviations: IPA, isopropanol; Et-OH, ethanol; MEO, methanol; 1,4DO, 1,4-dioxane; DMSO, dimethyl sulfoxide; DMF, dimethylformamide; ACE, acetone; ACN, acetonitrile; THF, tetrahydrofuran; 2-ME, 2-mercaptoethanol.

The Lac2 showed an optimal temperature of 70 °C and an optimal pH of 2.5 for ABTS oxidation. The enzyme remained stable at temperatures below 60 °C and within a pH range of 2.5–7.0 (Figure 4a). The half-lives of recombinant Lac2 at 50 °C, 60 °C, and 65 °C were 8.7 h, 51 min, and 17 min, respectively (Supplementary Figure S2). We also compared these properties with those of the native laccase from *H. abietinus* and found that, while the activity profiles were similar, the recombinant enzyme exhibited reduced thermal stability compared with the native enzyme.

The effects of nine water-miscible organic solvents on the activity and stability of purified Lac2 were evaluated, as these solvents are commonly used in industrial processes. Lac2 retained activity in the presence of 10% (*v*/*v*) of all solvents tested. At 50% solvent concentration, its activity decreased to 25–50%. When the concentration was increased to 80%, the enzyme maintained 20–39% of its activity in ethanol, isopropanol, methanol, and 1,4-dioxane (Figure 4c). After incubation with 50% organic solvents for 1–5 h, Lac2 showed only a slight loss of activity. Among all solvents tested, only THF completely inhibited the enzyme (Figure 4d). Similar results were observed with syringaldazine, a substrate soluble in organic solvents (Supplementary Figure S3).

The sensitivity of Lac2 to several known laccase inhibitors and metal ions was also assessed. The recombinant enzyme was strongly inhibited by NaN_3_, L-cysteine, and SDS, while EDTA had no detectable inhibitory effect (Figure 4e). Regarding metal ions, Lac2 was strongly inhibited by 10 mM Hg^2+^ (99% inhibition), Ag^2+^ (77%), and Fe^3+^ (49%). In contrast, Mn^2+^ and Zn^2+^ caused only minor inhibition, whereas Pb^2+^, Cu^2+^, Ca^2+^, and Mg^2+^ slightly stimulated the enzyme (Figure 4f).

## 3. Discussion

Ever since they were first discovered in the sap of the Japanese lacquer tree by Hikorokuro Yoshida in 1883 [42], laccases have remained a prominent focus of research, with around 1000 articles published on them each year over the past five years (results from the Web of Science Core Collection) [43]. This is not surprising because laccases are widely used in the textile, food, and pharmaceutical industries, as well as in many other fields. Laccases are also utilized in the research and development of biofuels and biochemicals. Gene cloning facilitates sequence-level modifications that increase functional adaptability, while recombinant protein synthesis provides an efficient and reproducible route for producing the resulting proteins. Therefore, in this research, Lac2 from *Hirschioporus abietinus* was cloned and heterologously expressed in *P. pastoris* cells. The amino acid sequence of the Lac2 was shown to exhibit high levels of homology with the other basidiomycetes, such as *Trametes versicolor* [44]. Comparison of Lac2 with its nearest structural homologue in the PDB showed a sequence identity of 72%. The highest degree of conservation of all laccases was observed in the copper-binding residues and the overall β-sheet cupredoxin fold. In contrast, the most variable regions corresponded to surface-exposed loops and the C-terminal region (Supplementary Figure S4). The occurrence of signal peptide indicated that C-terminal processing of Lac2 was taking place. This process has also been proposed for laccases from other basidiomycetes, such as *Lentinula edodes* [45], *Trametes* sp. [46], *Coprynus comatus* [47], and others. The extracellular detection of recombinant laccase utilizing its own signal peptide indicated its functionality in vivo. Comparison of the genomic and cDNA sequences of *lac2* showed the presence of 17 introns varying in size from 50 to 70 bp. Typically laccase genes contains 2–15 introns [48,49,50], although laccase genes containing up to 23 introns have been described [51].

Whereas native laccase purified from the culture medium of *H. abietinus* migrates as a 63 kDa band on SDS/PAGE, the recombinant protein has an apparent molecular weight of 66 kDa. Laccases are known to be secreted glycoproteins, and four putative glycosylation sites (Asn-X-Thr/Ser) were observed in the predicted amino acid sequence of the *H. abietinus* laccase. Recent studies demonstrate differences in molecular weight between proteins secreted by the natural host and the corresponding recombinant proteins expressed in *P. pastoris*. The obtained higher molecular weight protein band is likely due to hyper-glycosylation, since it is known that *P. pastoris* produces glycoproteins with chains of 8–14 mannose residues [52,53]. A similar effect of hyper-glycosylation was observed in the case of other laccases expressed in *P. pastoris* [54,55,56]. Although this change did not affect the activity of recombinant Lac2, it may be responsible for the decrease in thermostability of the recombinant enzyme. Although glycosylation often provides enzymes with thermal stability, longer glycosides can sometimes interfere with their function. For example, they can destabilize local folding, increase the flexibility of surface loops, or disrupt stabilizing salt bridges. Such a case was observed in alpha-amylase expressed in *P. pastoris* [57].

An earlier study of laccase expression in *P. pastoris* and *S. cerevisiae* demonstrated the importance of pH, temperature, methanol concentration, and availability of cofactors for the level of laccase expression [58,59,60]. In the case of recombinant Lac2 laccase, the expression was mostly affected by temperature. Reducing it from 30 °C to 20 °C increased laccase synthesis by 4-fold; a similar situation was observed in the case of *Trametes versicolor* laccase [61]. The pH of the medium also influenced the accumulation of Lac2; the highest level of protein synthesis occurred at a pH of 6. Unlike many recombinant laccases derived from *P. pastoris*, the best induction of Lac2 occurred at elevated methanol concentration (3%); for the other laccases expressed in *P. pastoris,* the optimal concentration of methanol was 0.5 to 1% [62,63,64]. The presence of cofactor in the medium also affected the synthesis of recombinant laccase. The absence of CuSO_4_ in the medium resulted in a four-fold decrease in activity. Similar results have been observed in other cases of recombinant laccase expression in *Pichia pastoris*. It is suggested that if there is insufficient copper during expression, the protein folds incorrectly or remains in an inactive apo form because copper cofactors are not incorporated into the enzyme structure [58,65,66,67,68]. In our study, laccase activity was observed even without the addition of copper (although reduced). Since cultivation is carried out in a rich medium (e.g., ~0.5–5 µg Cu per gram of yeast extract), copper is incorporated into the enzyme during synthesis, enabling its activity. It is possible in heterologous expression to obtain laccase at a level close to the g/L [69]; *P. pastoris* usually achieves up to 500 mg/L [46,56,67,70]. In the case of Lac2, about 10,000 U/L was received, which corresponds to 16 mg/L.

The recombinant laccase Lac2 was purified using a protocol developed for the purification of native enzyme from *H. abietinus* [37]. Since recombinant yeast did not produce pigments and polysaccharides characteristic of *H. abietinus*, the purification process was easier. The purified Lac2 had characteristics typical for fungal laccases. It oxidized a range of substrates, including a complex phenol (syringaldazine), phenoxazine ring-containing compound (3-(10H-phenoxazin-10-yl)propanoic acid), and an inorganic compound (hexacyanoferrate(II)) (Table 1). Basidiomycete laccases share a conserved multi-copper oxidase core and have broad oxidative capacity, but they mainly differ in T1 copper redox potential, stability, substrate specificity, and glycosylation, which reflect adaptation to different ecological roles. When comparing Lac2 with the closest described laccases, differences in specific activities and stability are observed (Supplementary Table S1). Studies on several possible laccase inhibitors showed that EDTA, a metal ion chelator, was not an effective inhibitor of the purified Lac2 laccase, similar to the effect on laccase from *Trametes hirsute* [71] and *Coprinopsis cinerea* [72], whereas sodium azide, L-cysteine, and SDS strongly inhibited laccase activity. This is consistent with results observed for laccases from *Trichoderma harzianum* [73] and *Leucaena leucocephala* [74]. Lac2 was unaffected by most of the metal ions studied. However, 10 mM of Fe^3+^, Ag^+^, and Hg^2+^ repressed laccase activity, suggesting that they may occupy Cu^2+^ binding sites and alter enzyme conformation.

The standard redox potential of its redox centre is the key characteristic of laccase. The structural reasons for the variability in redox potentials among laccases from different sources have not yet been fully elucidated. The redox potentials of the T1 site of laccases from different organisms vary over a wide range from 0.43 V (*Rhus vernicifera* [75] and *Coprinus cinereus* [76]) to 0.810 V (*Trametes hirsuta* 072 [77]) and the most critical factor determining the redox potential is the coordination sphere of the T1 copper and the more distant environment of the T1 site (the second coordination sphere) [78]. The presence of a phenylalanine at the axial position might be responsible for the high redox potential, leucine—medium redox potential, and methionine—low redox potential. The existence of the leucine–glutamate—alanine (LEA) tripeptide found in the T1 pocket seemed to correlate with high redox potential, whereas the presence of the valine–serine–glycine tripeptide (VSG) correlates with low redox potential. Lac2 had a high redox potential as well as leucine in the axial position and the LEA triplet in the T1 pocket. The presence of the LEA triplet does not contradict the high potential. On the other hand, the presence of leucine may indicate a lower potential than that of other laccases with a high redox potential, such as *Coriolus hirsutus* [79], *Trametes versicolor* [80], *Trametes villosa* [81], and *Pycnoporus* sp. [5], which have phenylalanine at the axial position.

Organic solvents usually decrease laccase activity, and only a few enzymes remain active in water–solvent mixtures. Lac2 maintained its activity in the presence of 10–50% of various organic solvents and was relatively stable in solutions supplemented with 50% organic solvents. Similar activity results toward organic solvents were obtained for laccases of *Chalara paradoxa* [82], *Polyporus pinsitus* [31], and *T. versicolor* [28]. *P. pinsitus* laccase retained 20% of its initial activity in the presence of 50% of 1,4-dioxane and isopropanol during 5 h incubation and was completely inhibited by acetonitrile [31], and *T. versicolor* laccase was completely inhibited by 30% of acetonitrile [83]. Enzymatic reactions in compatible organic solvents enable access to some insoluble substrates, which may aid the detoxification of several persistent organic pollutants [84]. The ability of Lac2 to retain its activity in organic solvents as well as in the presence of some metal ions makes it suitable for certain biotechnological applications.

In conclusion, characterization of the extracellular high-redox potential laccase Lac2 from fungus *Hirschioporus abietinus* undoubtedly allows enlargement of a toolbox of copper-containing oxidoreductases, which have a potential for the development of new biosensors and biocatalytic processes. In addition, availability of the recombinant version of the Lac2 encoding gene opens a direct way for further tailoring of the enzyme, particularly concerning substrate scope and improvement of protein stability.

## 4. Materials and Methods

### 4.1. Chemicals

ABTS, 2,6-DMP, SGZ, PZ, and metal salts (AgNO_3_, Ca(NO_3_)_2_, Co(NO_3_)_2_, CuSO_4_, MgSO_4_, MnSO_4_, ZnSO_4_, FeCl_3_, HgCl_2_, Pb(CH_3_COO)_2_, and NaN_3_) were all purchased from Sigma–Aldrich (Buchs, Switzerland). 3-(10*H*-phenoxazin-10-yl)propanoic acid (PPA) was synthesized as described previously [85], and potassium hexacyanoferrate (II) was obtained from Reachim (Moscow, Russia). PCR Master Mixes (2X); restriction endonucleases; T4 DNA ligase; pTZ57R/T vector; pPink-HC vector; PichiaPinkTM Strain1, Strain2, Strain3, and Strain4; and the First Strand Synthesis Kit were purchased from Thermo Scientific (Vilnius, Lithuania). All other chemicals were of standard reagent grade. All electrodes were from PalmSens BV (Houten, The Netherlands).

### 4.2. Isolation of Genomic DNA and Total RNA

To isolate genomic DNA and RNA, mycelia of *H. abietinus* were grown in a liquid medium consisting of 7 g/L malt extract, 5 g/L yeast extract, 2 g/L KH_2_PO_4_, 0.5 g/L MgSO_4_, 2 g/L glucose, and 0.2 mM CuSO_4_ with agitation at 30 °C for 3 days. The mycelia were harvested by centrifugation and genomic DNA was extracted using the ZR Fungal/Bacterial DNA Kit, Zymo Research (Irvine, CA, USA) and total RNA using the ZR Soil/Fecal RNA MicroPrep Kit, Zymo Research (Irvine, CA, USA).

### 4.3. Cloning of Laccase Gene

The degenerate primers (Table 3) were designed according to the conserved amino acid sequences of the copper-binding regions (Cu1F, Cu2R, and Cu3R for I, II, and III, respectively) and according to the peptide sequence of laccase from *H. abietinus* (Lac2R). Degenerate PCR was performed using the genomic DNA of *H. abietinus* Lac2 as the template. Based on the partial sequence obtained, inverse PCR primers were constructed. The chromosomal DNA was fully digested with KpnI, EcoRI, and HindIII, self-ligated by T4 DNA ligase, and then used as templates. The flanking sequences of the laccase DNA fragments were amplified using Maxima Hot Start PCR Master Mix with inverse primers of RevLac21FI and RevLac21R1 (1.5 kb of Kpn fragment), RevLac21F2 and RevLac21R2 (2.4 kb EcoRI fragment), and RevLac23F2 and RevLac23R2 (1.5 kb HindIII fragment). The PCR products were ligated into the pTZ57R/T vector and sequenced. The sequence of the laccase gene was assembled by the VectorNTI 9 programme.

### 4.4. RT-PCR and Cloning of Laccase cDNA

The full-length cDNA of Lac2 was obtained from an oligo(dT)18 primer using the RevertAid Premium First Strand Synthesis kit and the RNA of *H. abietinus*. According to the defined sequences of the laccase structural gene, primers LacRNApilF1 (matching the start codon ATG region) and LacRNApilR3 (matching the sequence immediately downstream of the stop codon TAA) were designed (Table 3). The full-length laccase gene was amplified from cDNA using DreamTag^TM^ PCR Master Mix. The obtained 1617 bp fragment was cloned into the pTZ57R/T vector and sequenced.

### 4.5. Construction of Expression Vectors for Pichia pastoris

The cDNA encoding mature *H. abietinus* laccase was amplified by PCR using primers LacEcoF and LacNaeR (Table 3). The PCR product was digested with EcoRI and NaeI restriction endonucleases and cloned into the pPink-HC vector through EcoRI–StuI restriction sites. The cloned gene sequence was confirmed by sequencing the insert of the constructed plasmid pHC-lac2-4. The construct and the control vector pPink-HC were linearized with BcuI and transformed into the competent PichiaPinkTM Strain1, Strain2, Strain3, or Strain4. Electrocompetent *P. pink* cells were prepared by 1 M sorbitol. A total of 5 µg of pHC-lac2-4 plasmid was used for each transformation reaction. Electroporation was carried out in an Eppendorf electroporation system with a 0.1 cm cuvette (BIORAD Hercules, CA, USA). After electroporation, YPDS medium was added and the sample was incubated for 4 h at 30 °C. The culture was then spread onto a PAD (pichia adenine dropout) agar plate and incubated for 3–5 days at 30 °C. White colonies were selected. To check for secreted laccase activity in liquid culture, a single transformant containing the laccase gene in genomic *P. pastoris* DNA (verified by PCR using LacEcoF and LacNaeR primers) was grown in 20 mL BMGY medium in a 125 mL Erlenmeyer flask overnight at 30 °C in a shaking incubator at 200 rpm. The cells at an optical density of 1.2–1.8 were centrifuged at 4000× *g* for 2 min, and the cell pellets were suspended in 10 mL BMMY media (pH 6.0) containing 0.2 mM CuSO_4_. The cultures were grown at 30 °C with shaking at 200 rpm for 5 h, then 1% (*v*/*v*) methanol was added, and cells were grown for 48 h. Then the cultures were centrifuged at 3000× *g* for 5 min. The secretion of laccase activities in cultures was measured. Afterwards, several different incubation temperatures (20, 25, and 30 °C), pHs (3–7), and concentrations of methanol (ranging from 0.5% to 10%) were set and tested. In each case, all factors remained constant (25 °C, pH 6, and 1% methanol) except the one under investigation.

To purify the laccase, the strain was first cultured overnight in 50 mL of BMGY medium in a 125 mL Erlenmeyer flask at 25 °C with shaking at 200 rpm. Cells were harvested at an optical density of 1.2–1.8 by centrifugation at 4000× *g* for 5 min, then resuspended in 200 mL of BMMY medium (pH 6.0) supplemented with 0.2 mM CuSO_4_. Cultures were incubated at 20 °C with shaking at 200 rpm for 5 h, after which methanol was added to a final concentration of 3% (*v*/*v*). The cultures were then maintained under these conditions for 12 days.

### 4.6. Protein Purification

Recombinant laccase from PichiaPinkTM Strain1 was purified as described previously [37]. Briefly, the centrifuged and concentrated (polyethersulfone membrane, 10 kDa cut-off) culture fluid was dialyzed with a 10 mM potassium phosphate buffer solution (pH 7.0). The purification scheme consisted of three stages: 1. DEAE-Toyopearl 650 M column (ToyoSoda, Tokyo, Japan) (elution with a linear gradient of 10–500 mM potassium phosphate buffer); 2. Phenyl Sepharose 6 Fast Flow column (Cytiva, Marlborough, MA, USA) (elution with 10 mM potassium phosphate buffer (pH 7.0) and a gradient from 1.5 to 0 M ammonium sulfate); 3. Source 15Q column (Cytiva, Marlborough, MA, USA) (elution using a 10–500 mM potassium phosphate gradient (pH 7.0)). The purified fractions were concentrated and stored at −20 °C.

### 4.7. Assay of Laccase Activity

The activity of laccase was measured using 2,2′-azino-bis(3-ethylbenzothiazoline-6-sulphonic acid) (ABTS) as a substrate as described previously [86].

### 4.8. Characterization of Recombinant Laccase

Protein concentration was determined by Bradford assay with bovine serum albumin (BSA) as a standard. The effect of pH on laccase activity was evaluated at 30 °C at a pH range of 2.5–7.0, using 0.1 M sodium citrate buffer. Further study on the pH stability of the recombinant laccase was carried out by pre-incubating the enzyme solutions in 0.1 M sodium citrate buffer at different pH levels (2.5–7.0) in the absence of substrate at 30 °C for 1 h. The temperature dependence of recombinant laccase activity was evaluated by measuring ABTS oxidation under the previously described conditions at temperatures between 30 and 75 °C. The thermal stability of the purified enzyme was estimated as described previously [86].

For determination of K_M_ and V_max_, the kinetic curves were recorded at the wavelength corresponding to the maximum of absorbance as described in [87]. The Lineweaver–Burk plot method was used to determine the K_M_ and V_max_ values of the purified laccase. Decreasing fluorescence intensity during 1-naphthol oxidation was registered at 460 nm when excitation was 320 nm. The concentrations of oxidation products were calculated using a fluorescence intensity coefficient determined from the calibration curves. The impact of various metal ions, inhibitors, and organic solvents on laccase activity was evaluated at 30 °C and pH 3.0 using the ABTS assay after a 10 min incubation period. The stability in the presence of an organic solvent was estimated by incubating it in a 50% solvent solution for 1–5 h before conducting the activity assay. Results are shown as the mean ± SD of three reactions.

### 4.9. Determination of the Redox Potential of the T1 Site

A common T1 copper site redox titration method was used exploiting reduced mediator K_4_[Mo(CN)_8_] as previously described [20]. Briefly, spectroelectrochemical titrations were performed in a 0.1 mL cell, as previously described [88], using a gold working electrode and a platinum counter electrode in conjunction with an Ag/AgCl/KCl/sat reference electrode (0.2 ± 0.002 V vs. NHE). Laccase (100 µL at 10.38 mg/mL) was added to a solution of 0.5 mM mediator in 10 mM phosphate buffer solution (pH 7.0), which was then equilibrated for 40 min. Potentials were then applied from 0.5 to 1 V in 0.05 V steps, after which the scan was reversed. Absorbance spectra were recorded at each step with minimal light exposure and it was assumed that oxygen had been depleted. The T1 redox potential was determined from the dependence of the absorbance at 602 nm on potential, using the Nernst Equation (1) with normalized absorbance values:

(1) $$E_{i}=E_{redox}+\frac{R\times T}{n_{e}\times F}ln\left(\frac{{rAbs}_{i}}{1-{rAbs}_{i}}\right)$$

where *E_i_*—applied potential at the *i* step, *E_redox_*—redox potential of T1 site, *n_e_*—number of electrons involved in the redox process, *rAbs_i_*—relative absorption at the *i* step, calculated as (2):

(2) $${rAbs}_{i}=\frac{{Abs}_{i}-{Abs}_{min}}{{Abs}_{max}-{Abs}_{min}}$$

where *Abs_i_*—absorbance value at the *i* step, *Abs_max_* and *Abs_min_*—maximal and minimal absorbance values.

### 4.10. Bioinformatic Analysis

Sequence homology of Lac2 with known laccases was analyzed using BLAST (http://blast.ncbi.nlm.nih.gov/Blast.cgi/, accessed on 10 November 2025 (BLAST+ version 2.17.0)). Molecular weight was predicted with Vector NTI, N-glycosylation sites (Asn-X-Ser/Thr) with NetNGlyc 1.0 (http://www.cbs.dtu.dk/services/NetNGlyc/, accessed on 25 October 2025), and the signal peptide with Signal 3L (http://www.csbio.sjtu.edu.cn/bioinf/Signal-3L/#, accessed on 20 October 2025). The 3D structure was modelled using AlphaFold2 [89] and visualized by ChimeraX [90].

### 4.11. Phylogenetic Analysis

The sequences of laccases for phylogenetic analysis were retrieved from the UniProtKB/SwissProt and PDB protein database. The evolutionary history was inferred using the Neighbour-Joining method [91]. The total branch length was 3.57855447. The percentage of replicate trees in which the associated taxa clustered together in the bootstrap test (2000 replicates) is shown next to the branches [92]. The tree is drawn to scale, with branch lengths measured in the same units as the evolutionary distances used to construct the phylogenetic tree. The evolutionary distances were calculated using the p-distance method [93] and are expressed in units of amino acid substitutions per site. The analysis included 34 amino acid sequences. All positions with gaps and missing data were removed. The final dataset consisted of 209 positions in total. Evolutionary analyses were performed in MEGA7 [94]. The sequences obtained from the present study were deposited in the GenBank database under the accession number MN180198.1.

## Figures and Tables

**Table 1:** Substrate specificity and apparent kinetic constants of Lac2.

Substrate	K_M_ µM	k_cat_, s^−1^	k_cat_/K_M_, µM^−1^·s^−1^	Optimal pH
PPA	3.6 ± 0.1	82.0 ± 5.0	22.8 ± 0.6	3.0
K_4_Fe(CN)_6_	5.4 ± 1.0	120.0 ± 5.0	22.2 ± 4.0	3.0
SGZ	20.0 ± 2.0	6.1 ± 0.4	0.3 ± 0.02	5.5
ABTS	42.0 ± 7.0	87.0 ± 6.0	2.1 ± 0.3	3.0
1-naphthol	119.1 ± 20.7	22.6 ± 2.7	0.19 ± 0.02	5.5
PZ	120 ± 10	27.0 ± 1.0	0.2 ± 4.0	5.0
2,6-DMP	180 ± 60	18.8 ± 5.0	0.1 ± 0.01	5.0
HQ	389.0 ± 102	22.0 ± 3.0	0.056 ± 0.01	5.5
CAT	620.0 ± 246	65.0 ± 17.0	0.10 ± 0.01	5.5

ABTS, 2,2′-Azino-bis(3-ethylbenzothiazoline-6-sulfonate); 2,6-DMP, 2,6-dimethoxyphenol; SGZ, 4-hydroxy-3,5-dimethoxybenzaldehyde azine; PZ, promazine hydrochloride; PPA, 3-(10*H*-phenoxazin-10-yl)propanoic acid; K_4_Fe(CN)_6_, potassium hexacyanoferrate (II); HQ, hydroquinone; CAT, 1,2-dihydroxybenzene.

**Table 2:** Estimated redox potential of the T1 site of Lac2 and the number of electrons involved in the redox process.

Scan	E_redox_ (V vs. NHE)	n_e_
Oxidative scan	0.709 ± 0.004	1.161 ± 0.007
Reductive scan	0.720 ± 0.007	0.688 ± 0.007
Average	0.714 ± 0.12	0.92 ± 0.24

**Table 3:** Oligonucleotide primers used in this study.

Primer	Nucleotide Sequence 5′–3′
Cu1F	CAYTGGCAYGGNTTYTTY
Cu2R	GRCTGTGGTACCAGAANGTNCC
Cu3R	TGNCCRTGNARRTGNANNGGRTG
Lac2F	GGNACNWNNTGGTAYCAYWSNCA
LacRNApilF1	TGCATAGGTCACTGCTCATCATGCGAAG
LacRNApilR3	TCACTCGAAAGCTGTGTCTGGATCGTTTG
RevLac21FI	GTTAATATTCAGGATGTATCAC
RevLac21R1	CTGAATGTGCGTCTCAATCAG
RevLac21F2	CGAAGTGTTAATATTCAGGAT
RevLac21R2	GTGGGCTGAATGTGCGTCTCA
RevLac23F2	CTAGTCTTTAGCCAATAATAC
RevLac23R2	AGTCTGCCCTCGAACAAGGTA
LacEcoF	TCGAATTCGAAACGATGCGAAGCTTTGCC
LacNaeR	TTGCCGGCTCACTCGAAAGCTGTGTCTG

## Data Availability

The original contributions presented in the study are included in the article; further inquiries can be directed to the corresponding author.

## Supplementary Materials

The following supporting information can be downloaded at: https://www.mdpi.com/article/10.3390/molecules31030458/s1, Figure S1: Effect of copper on the activity of secreted Lac2. (a) Influence of copper concentration on laccase yield in the culture medium after 6 days of growth at 25 °C in BMMY (pH 6) medium. CuSO_4_ was added at the beginning of the induction phase. (b) Activity of secreted laccase following copper supplementation. After 3 days of growth, the culture medium was centrifuged, supplemented with 0.2 μM CuSO_4_, and incubated at 22 °C. Open circles–medium without copper and closed circles–medium with 0.2 mM copper. The data represent two independent experiments; Figure S2: Lac2 activity and stability in the presence of organic solvents. (a) Relative activity of Lac2 in the presence of various organic solvents at 10 and 50% (*v*/*v*). (b) Stability of Lac2 after incubation in 50% of organic solvents for 1, 3, and 5 h prior to activity assay. The effects of organic solvents on the activity of laccase were evaluated at 22 °C using 20 μM syringaldazine as a substrate, 100 mM sodium acetate buffer pH 5.0, and 0.02 mg of enzyme in a 1 mL reaction mixture by measuring the initial linear slope at A525 nm for 1 min. The values are presented as the mean ± SD of triplicate reactions. Prior to measurements, we checked the substrate’s response to the organic solvent. Enzyme activity or stability in a buffer without an organic solvent is 100%. IPA, isopropanol; Et-OH, ethanol; MEO, methanol; 1,4DO, 1,4-dioxane; DMSO, dimethyl sulfoxide; DMF, dimethylformamide; ACE, acetone; ACN, acetonitrile; THF, tetrahydrofuran; Figure S3: Termostability of Lac2 in 50 °C (a), 60 °C (b), and 65 °C (c). A total of 0.2 mg/mL of Lac2 in 20 mM sodium citrate buffer (pH 3) was incubated at the appropriate temperature at different time intervals prior to the activity assay with ABTS as a substrate; Figure S4: Comparison of Lac2 and *R. microporus* (1V10 PDB) laccase. Left–tertiary structure of Lac2 (light blue) and *R. microporus* laccase (gold). Right–sequence alignment of Lac2 (QID90256.1) and *R. microporus* laccase (1V10_A). Sequence alignment was performed with ClustalW and analyzed with Espript3.0. Tertiary models were built with AlfaFold2; Table S1: Biochemical characteristics of Lac2 (*Hirschioporus abietinus*) and closely related laccases [95,96,97,98,99].

## Abbreviations

The following abbreviations are used in this manuscript:

ABTS	2,2′-Azino-bis(3-ethylbenzothiazoline-6-sulfonate)
2,6-DMP	2,6-Dimethoxyphenol
SGZ	4-Hydroxy-3,5-dimethoxybenzaldehyde azine
PZ	Promazine hydrochloride
PPA	3-(10*H*-phenoxazin-10-yl)propanoic acid
K_4_Fe(CN)_6_	Potassium hexacyanoferrate (II)
HQ	Hydroquinone
CAT	1,2-Dihydroxybenzene
IPA	Isopropanol
Et-OH	Ethanol
MEO	Methanol
1,4DO	1,4-Dioxane
DMSO	Dimethyl sulfoxide
DMF	Dimethylformamide
ACE	Acetone
ACN	Acetonitrile
THF	Tetrahydrofuran
2-ME	2-Mercaptoethanol
