# Supplementary material for: Hirschioporus abietinus Laccase: Cloning, Heterologous Expression, Characterization and Solvent Tolerance Evaluation
Source: Molecules. 2026 Jan 28;31(3):458. doi: 10.3390/molecules31030458 (PMC12899955; doi:10.3390/molecules31030458)
Supplement: Supplementary file 1 [file molecules-31-00458-s001.zip › molecules-4065490-supplementary.pdf]

Supplement

Hirschioporus abietinus Laccase: Cloning, Heterologous Expression, Characterization and Solvent -Tolerance Evaluation

Ingrida Radveikienė [1], Marius Dagys [1], Rita Meškienė [2], Rolandas Meškys [2], Regina Vidžiūnaitė [1], Vida Časaitė [2,*]

[1] Department of Bioanalysis, Institute of Biochemistry, Life Sciences Center, Vilnius University, Sauletekio Ave. 7, 10257 Vilnius, Lithuania; i.radveikiene@gmail.com (I.R.); marius.dagys@gmc.vu.lt (M.D.)

[2] Department of Molecular Microbiology and Biotechnology, Institute of Biochemistry, Life Sciences Center, Vilnius University, Sauletekio Ave. 7, 10257 Vilnius, Lithuania; rita.meskiene@bchi.vu.lt (R.M.); rolandas.meskys@bchi.vu.lt (R.M.)

* Correspondence: vida.casaite@bchi.vu.lt

Supplementary Table S1. Biochemical characteristics of Lac2 (*Hirschioporus abietinus*) and closly related laccases.

**Species**	**Km (ABTS) μM**	**T optimum, °C (ABTS)**	**Specific activity (U/mg)**	**pH optimum (ABTS)**	**Citation**
Trametes versicolor	11	50	91,443	3.0	[95]
Trametes hirsuta	20 ± 5	30	978	4–6	[96]
Pycnoporus sanguineus	32	70	32.9	3.0	[97]
Coriolus hirsutus	10–20	45	1,000–1,500	4.0	[98]
Trametes villosa	20–30	40–65	400–800	3.0	[99]
Rigidoporus microporus	177	55	-	2.4	[59]
Hirschioporus abietinus	42	70	700	3.0	This study

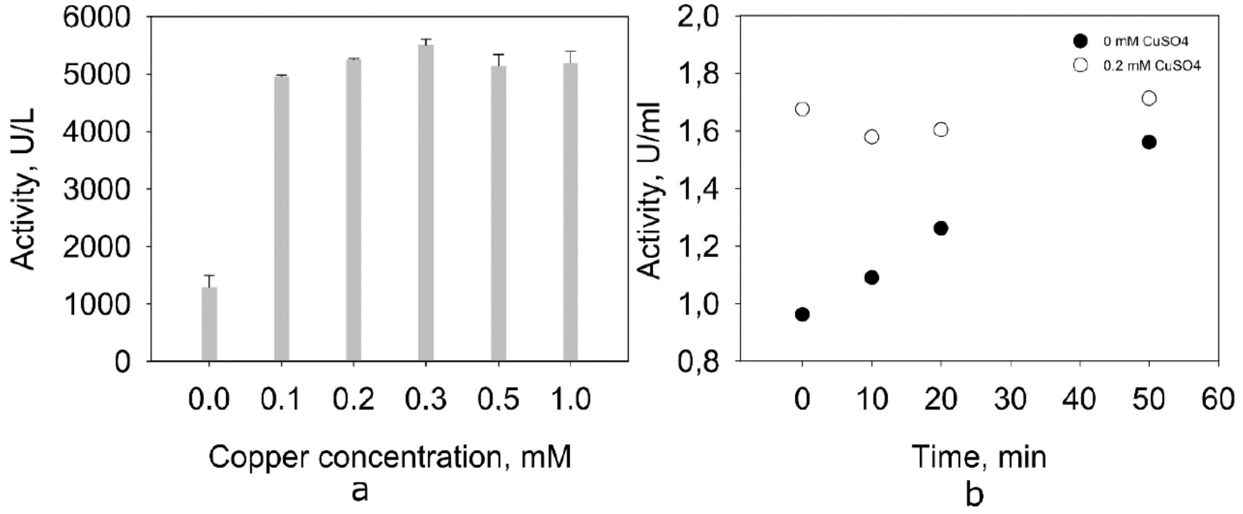

Supplementary Figure S1. Effect of copper on the activity of secreted Lac2. (a) Influence of copper concentration on laccase yield in the culture medium after 6 days of growth at 25 °C in BMMY (pH6) medium. $CuSO_4$ was added at the beginning of the induction phase. (b) Activity of secreted laccase following copper supplementation. After 3 days of growth, the culture medium was centrifuged, supplemented with 0.2 μM $CuSO_4$, and incubated at 22 °C. Open circles – medium without copper and closed circles – medium with 0.2 mM copper. The data represent two independent experiments.

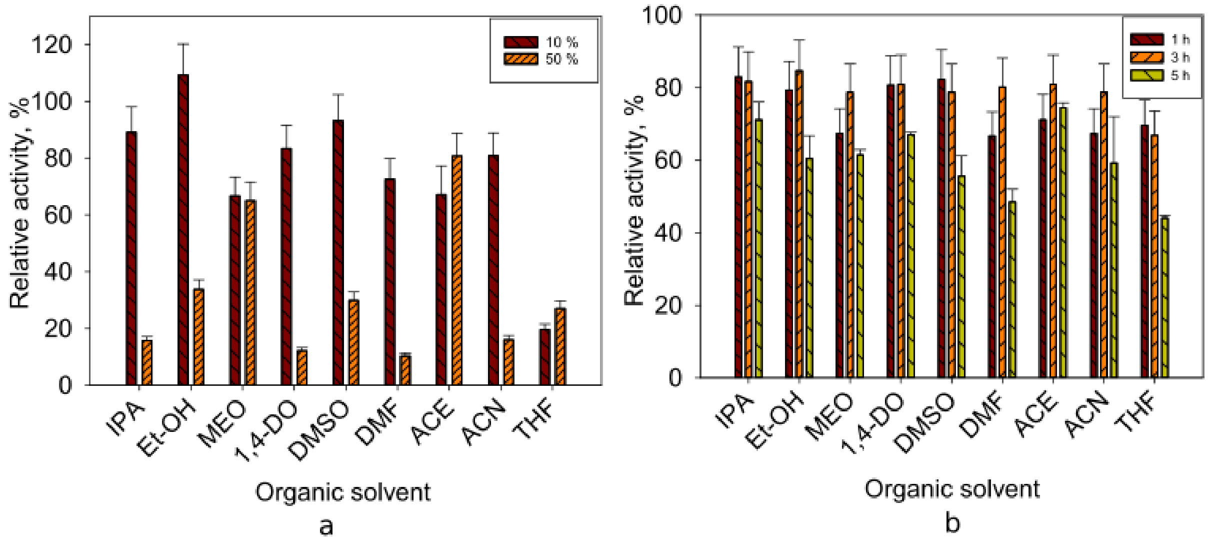

Supplementary Figure S2. Lac2 activity and stability in the presence of organic solvents. (a) Relative activity of Lac2 in the presence of various organic solvents at 10 and 50% (v/v). (b) Stability of Lac2 after incubation in 50 % of organic solvents for 1, 3, and 5 hours prior to activity assay. The effects of organic solvents on the activity of laccase were evaluated at 22 °C using 20 μM Syringalgazine as a substrate, 100 mM sodium acetate buffer pH 5.0, and 0.02 mg of enzyme in a 1 ml reaction mixture by measuring the initial linear slope at A_{525} nm for 1 min. The values are presented as the mean ± SD of triplicate reactions. Prior to measurements, we checked the substrate's response to the organic solvent. Enzyme activity or stability in a buffer without an organic solvent is 100 %. IPA, isopropanol; Et-OH, ethanol; MEO, methanol; 1,4DO, 1,4-dioxane; DMSO, dimethyl sulfoxide; DMF, dimethylformamide; ACE, acetone; ACN, acetonitrile; THF, tetrahydrofuran;

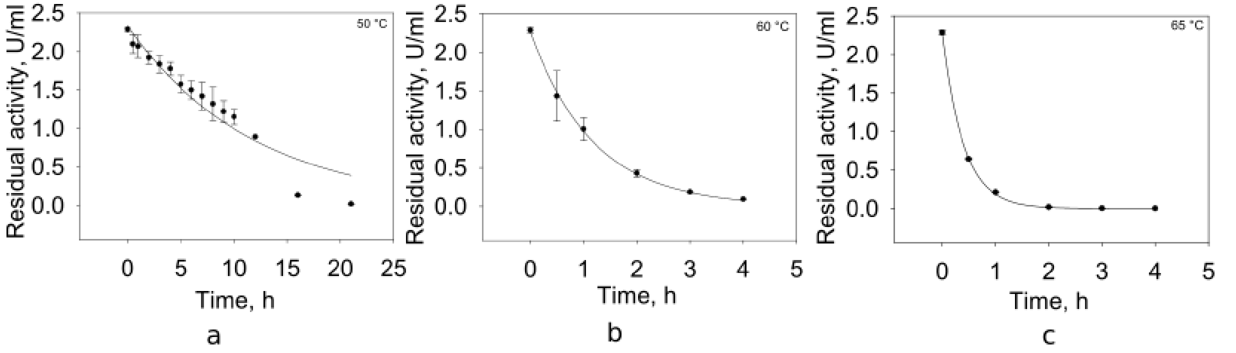

Supplementary Figure S3. Termostability of Lac2 in 50 °C (a), 60 °C (b), and 65 °C (c). A total of 0.2 mg/ml of Lac2 in 20 mM sodium citrate buffer (pH 3) was incubated at the appropriate temperature at different time intervals prior to the activity assay with ABTS as a substrate.

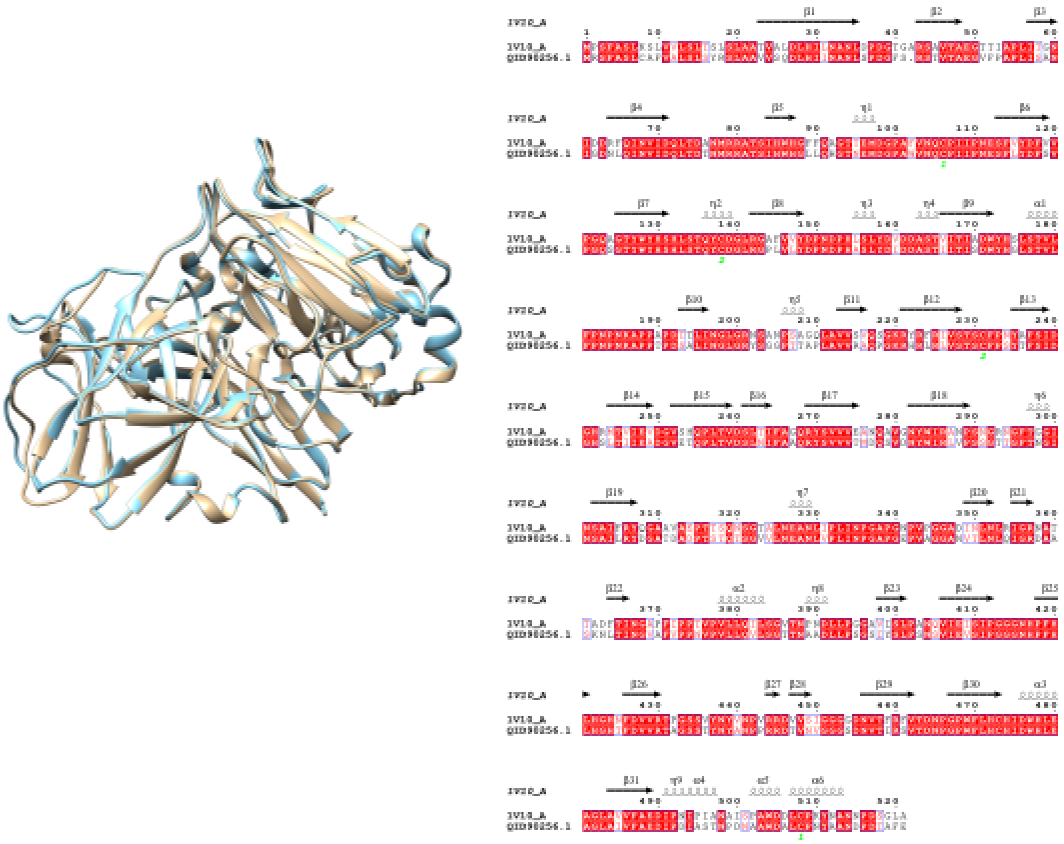

Supplementary Figure S4. Comparison of tertiary (left) and secondary (right) structure of Lac2 and *R. microporus* (1V10 PDB) laccase. Lac2 is light blue, and *R. microporus* laccase is gold. Sequence alignment of Lac2 (QID90256.1) and *R. microporus* laccase (1V10_A). Sequence alignment was performed with ClustalW and analyzed with Espript3.0. Tertiary models were built with AlfaFold2.

References:

59. Liu, W.; Chao, Y.; Liu, S.; Bao, H.; Qian, S. Molecular cloning and characterization of a laccase gene from the basidiomycete Fome lignosus and expression in Pichia pastoris. *Appl. Microbiol. Biotechnol.* **2003**, *63*, 174–81, doi:10.1007/s00253-003-1398-0.

95. Moon-Jeong, H.; Hyoung-Tae, C.; Hong-Gyu, S. Purification and characterization of laccase from the white rot fungus Trametes versicolor. *J. Microbiol.* **2005**, *43*, 555–560.

96. Navada, K.K.; Kulal, A. Kinetic characterization of purified laccase from Trametes hirsuta: a study on laccase catalyzed biotransformation of 1,4-dioxane. *Biotechnol. Lett.* **2021**, *43*, 613–626, doi:10.1007/S10529-020-03038-1.

97. Cheute, V.M.S.; Backes, E.; Pateis, V. de O.; de Oliveira Junior, V.A.; Uber, T.M.; dos Santos Filho, J.R.; dos Santos, L.F.O.; Castoldi, R.; de Souza, C.G.M.; Polonio, J.C.; et al. Optimization of Immobilization, Characterization, and Environmental Applications of Laccases from Pycnoporus sanguineus UEM-20. *Processes* **2025**, *13*, 1800, doi:10.3390/PR13061800/S1.

98. Lee, Y.J.; Shin, K.S. Purification and Properties of Laccase of the White-rot Basidiomycete Coriolus hirsutus. *J. Microbiol.* 1999, *37*, 148–153.

99. Yaver, D.S.; Xu, F.; Golightly, E.J.; Brown, K.M.; Brown, S.H.; Rey, M.W.; Schneider, P.; Halkier, T.; Mondorf, K.; Dalbøge, H. Purification, characterization, molecular cloning, and expression of two laccase genes from the white rot basidiomycete Trametes villosa. *Appl. Environ. Microbiol.* **1996**, *62*, 834–841, doi:10.1128/AEM.62.3.834-841.1996.
